# Supplementary material for: STAT1 potentiates oxidative stress revealing a targetable vulnerability that increases phenformin efficacy in breast cancer
Source: Nat Commun. 2021 Jun 3;12:3299. doi: 10.1038/s41467-021-23396-2 (PMC8175605; doi:10.1038/s41467-021-23396-2)
Supplement: Supplementary file 1 — Supplementary Information [file 41467_2021_23396_MOESM1_ESM.pdf]

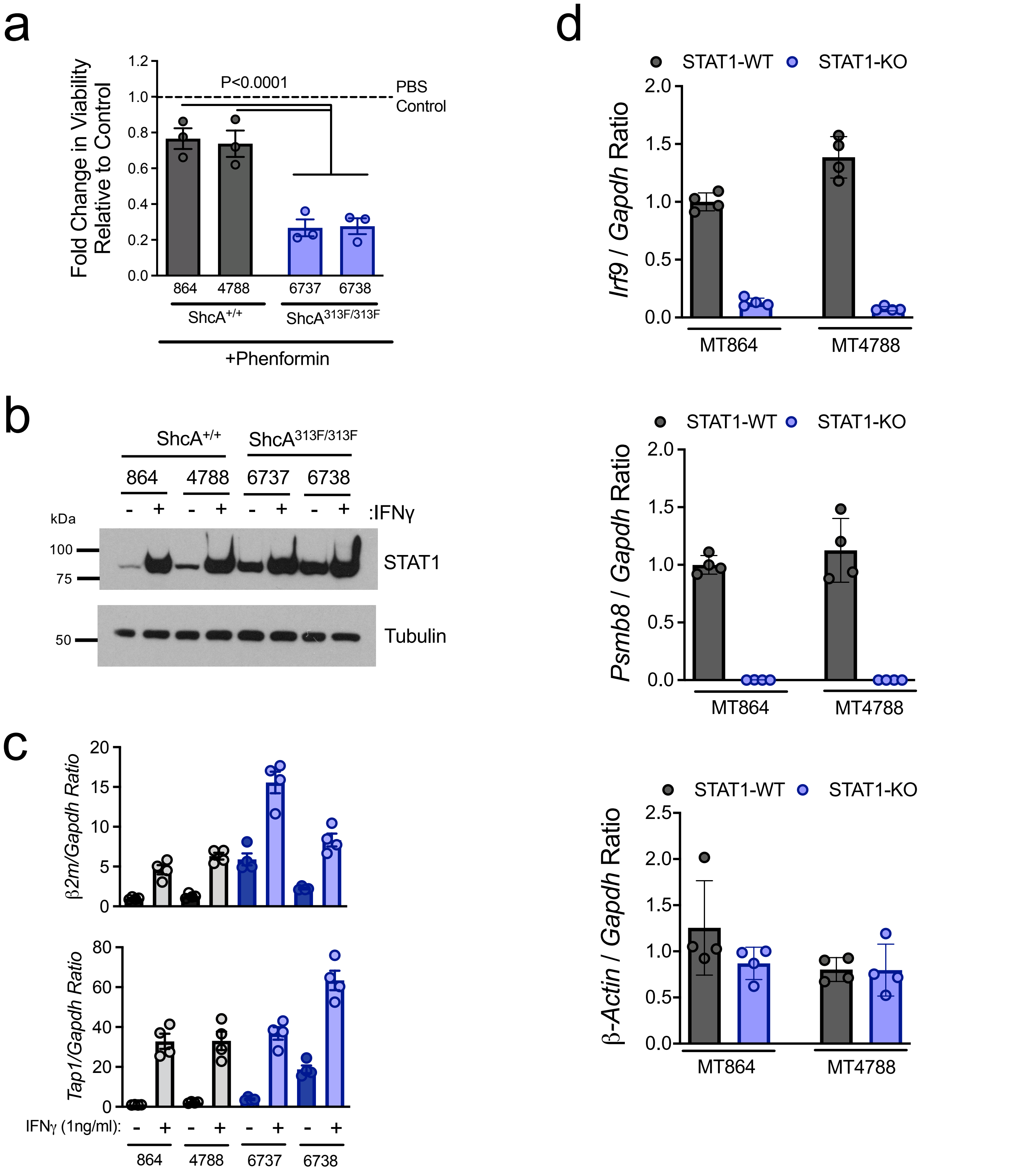

a
Fold Change in Viability Relative to Control
P<0.0001
PBS Control
864
4788
6737
6738
ShcA+/+
ShcA313F/313F
+Phenformin
b
ShcA+/+
ShcA313F/313F
864
4788
6737
6738
:IFNγ
kDa
100
75
50
STAT1
Tubulin
c
β2m/Gapdh Ratio
Tap1/Gapdh Ratio
IFNγ (1ng/ml):
864
4788
6737
6738
d
STAT1-WT
STAT1-KO
Irf9 / Gapdh Ratio
Psmb8 / Gapdh Ratio
β-Actin / Gapdh Ratio
MT864
MT4788

Supplementary Figure 1: Loss of ShcA Y313 phosphorylation in breast cancer cells increases phenformin sensitivity coincident with elevated STAT1 levels.
(a) Viability of ShcA wild-type and ShcA313F cells treated with phenformin (500 μM) for 48 hours. Data is shown as fold change in viability compared to PBS controls and is representative of n=3 independent experiments (mean of means ± SEM).
(b) STAT1 immunoblot analysis of control and IFNγ-treated ShcA wild-type and ShcA313F cells. Tubulin is used as the loading control. Blot is representative of n=3 independent experiments.
(c-d) RT-qPCR analysis of IFNγ target gene expression levels in the indicated cell lines compared to PBS control (n=4 technical repeats/condition). The data is shown as ratio with GAPDH used as loading control, mean ± SD.
P values were calculated using one-way ANOVA with a Tukey's posthoc test (panel a), see Figure.
See also Figure 1.

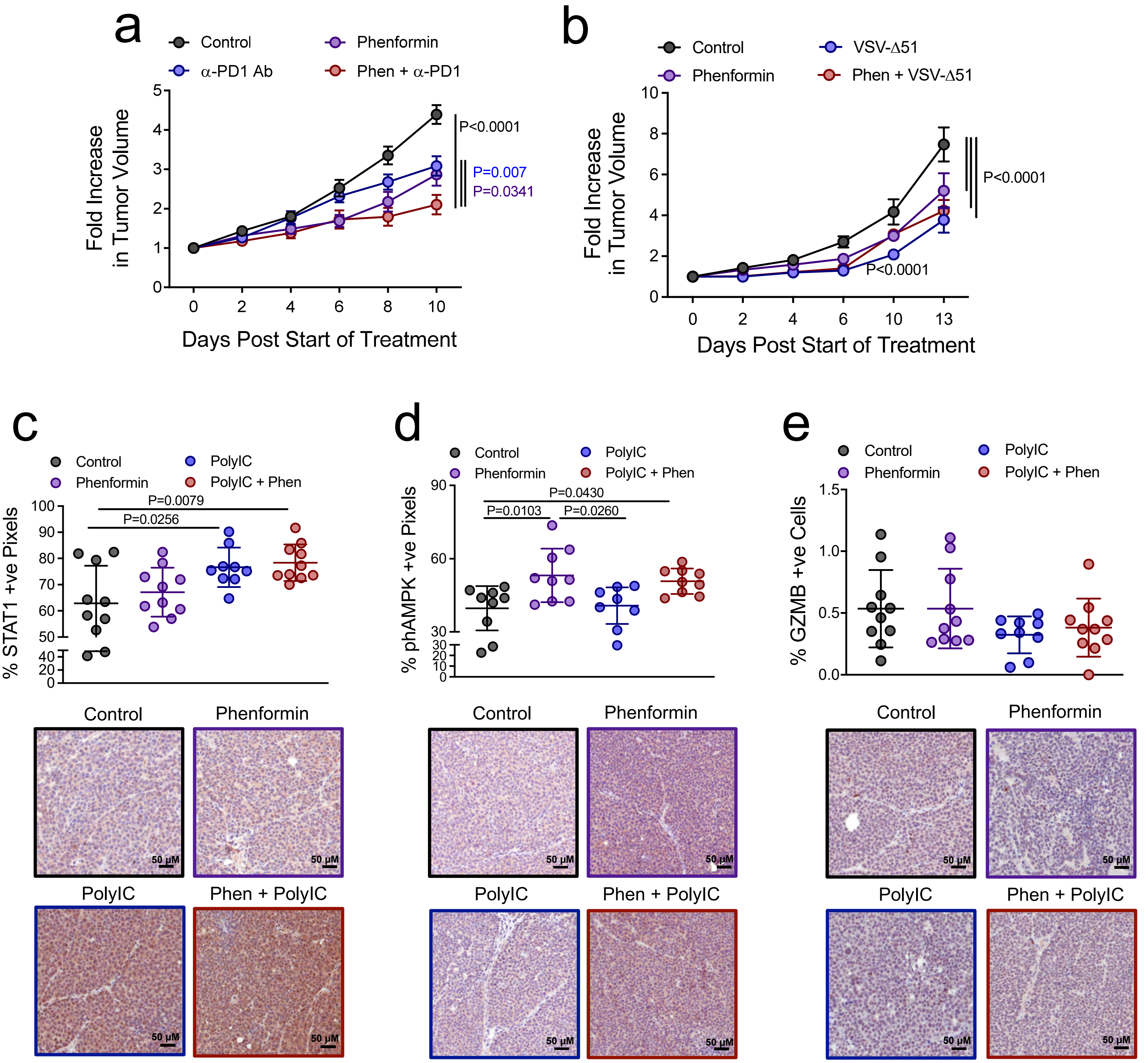

Supplementary Figure 2

Supplementary Figure 2: Immune-based therapies do not sensitize breast tumors to phenformin.

(a, b) MT4788 breast cancer cells were injected into the mammary fat pads of FVB mice. At 100 mm^3, tumor bearing mice were randomized and injected intraperitoneally with **(a)** phenformin (50 mg/kg daily), 100 µg anti-PD1 antibody (every 3 days), either alone or in combination. Isotype control IgG antibodies (100 µg every 3 days) were injected for the control and phenformin treatment groups.**(b)** Separate cohorts of mice were also treated with phenformin (50 mg/kg daily) or oncolytic virus VSV MΔ51 (two intra-tumoral injections of $1x10^7$ PFU administered 24 hours apart), alone or in combination. PBS was administered intraperitoneally and/or intra-tumorally for the control groups. Data is represented as fold increase in tumor volume relative to start of treatment (mean of means) ±SEM and is representative of **(a)** Control: n=9; anti-PD1: n=10; phenformin: 6; anti-PD1+Phen: n=6 tumors/group. P value were calculated compared to Phenformin + anti-PD1 group and are as follows: Control - P<0.0001 (black); Phenformin alone– P=0.0081 (purple) and anti-PD1 alone - P=0.0010 (blue) **(b)** Control: n=8; phenformin: n=7; VSV MΔ51: n=6; VSV+phenformin: n=8 tumors/group. P value were calculated compared to the control group and are as follows: P<0.001 compared to phenformin or VSV M Δ51, either alone or in combination.

(c-e) Immunohistochemical analysis of mammary tumors described in Figure 2c using **(c)** STAT1, **(d)** pAMPK and **(e)** Granzyme B-specific antibodies. The data is shown as the mean **(c, d)** % positive pixels or **(e)** % positive cells ±SEM and is representative of n=10 tumors/group, except PolyIC: n=9 tumors **(c)**; n=9 tumors/group, except PolyIC: n=8 tumors **(d);** n=10 tumors/group, except PolyIC: n=9 tumors **(e)**. Representative images are shown.

P values were calculated using two-way ANOVA with a Tukey's posthoc test (panels a, b) or one-way ANOVA using Tukey's posthoc test (panels c-e).

See also Figure 2.

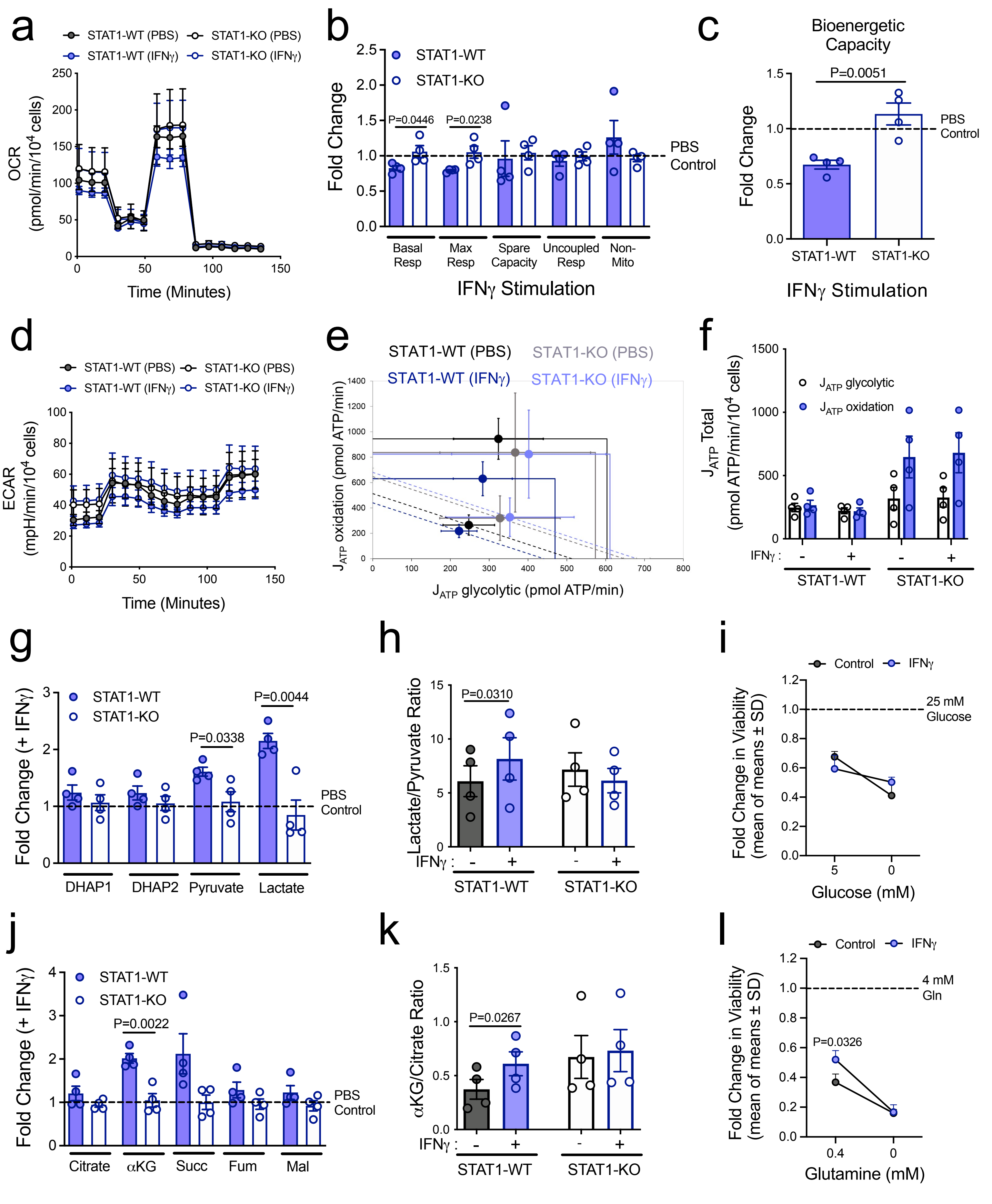

Supplementary Figure 3

Supplementary Figure 3: IFNγ signaling has no impact on mitochondrial ATP production in STAT1-deficient breast cancer cells.
(a) Seahorse measurements of the oxygen consumption rate (OCR) comparing STAT1-WT and STAT1-KO MT4788 cells after 24 hour of 1 ng/ml IFNγ or PBS treatment.
(b) Fold change in the rates of basal respiration, maximal respiration, spare capacity, uncoupled respiration and non-mitochondrial from samples in panel a, relative to their PBS controls.
(c) The fold change in total the bioenergetic capacity was determined from the samples described in panels a and b, relative to their PBS controls.
(d) Seahorse measurements of the extracellular acidification rate (ECAR) of the same cells in panel a.
(e, f) The total metabolic capacity and flexibility of breast cancer cells was determined by calculating the basal (point on dotted line) and maximal rates (point on solid line) of ATP production from glycolysis (J_{ATP} Glycolytic) and oxidative phosphorylation (J_{ATP} Oxidation),
(g) Fold change in steady state levels of glycolytic metabolites following 24hr IFNγ treatment of same cells as panel a, relative to their respective PBS controls.
(h) The lactate/pyruvate ratio was determined from the samples analyzed in panel g.
(i) Viability of MT4788 cells with IFNγ or PBS treatment upon glucose withdrawal (48 hours). Data is expressed as a fold change relative to their own treated controls in 25 mM glucose media, representative of n=3 independent experiments (mean of means) ±SEM.
(j) Fold change in steady state levels of citric acid cycle metabolites following 24 hr IFNγ treatment, relative to their respective PBS controls.
(k) α-ketoglutarate/citrate ratio was determined from the samples analyzed in panel j.
(l) Viability of MT4788 cells with IFNγ or PBS treatment upon glutamine withdrawal (48 hours). Data is expressed as a fold change relative to their own treated controls in 4 mM glutamine, and is representative of n=3 independent experiments (mean of means) ±SEM.
Unless specified above, all data elements are representative of n=4 independent experiments (mean of means) ±SEM. P values were calculated using two-tailed unpaired t-tests.
See also Figure 3.

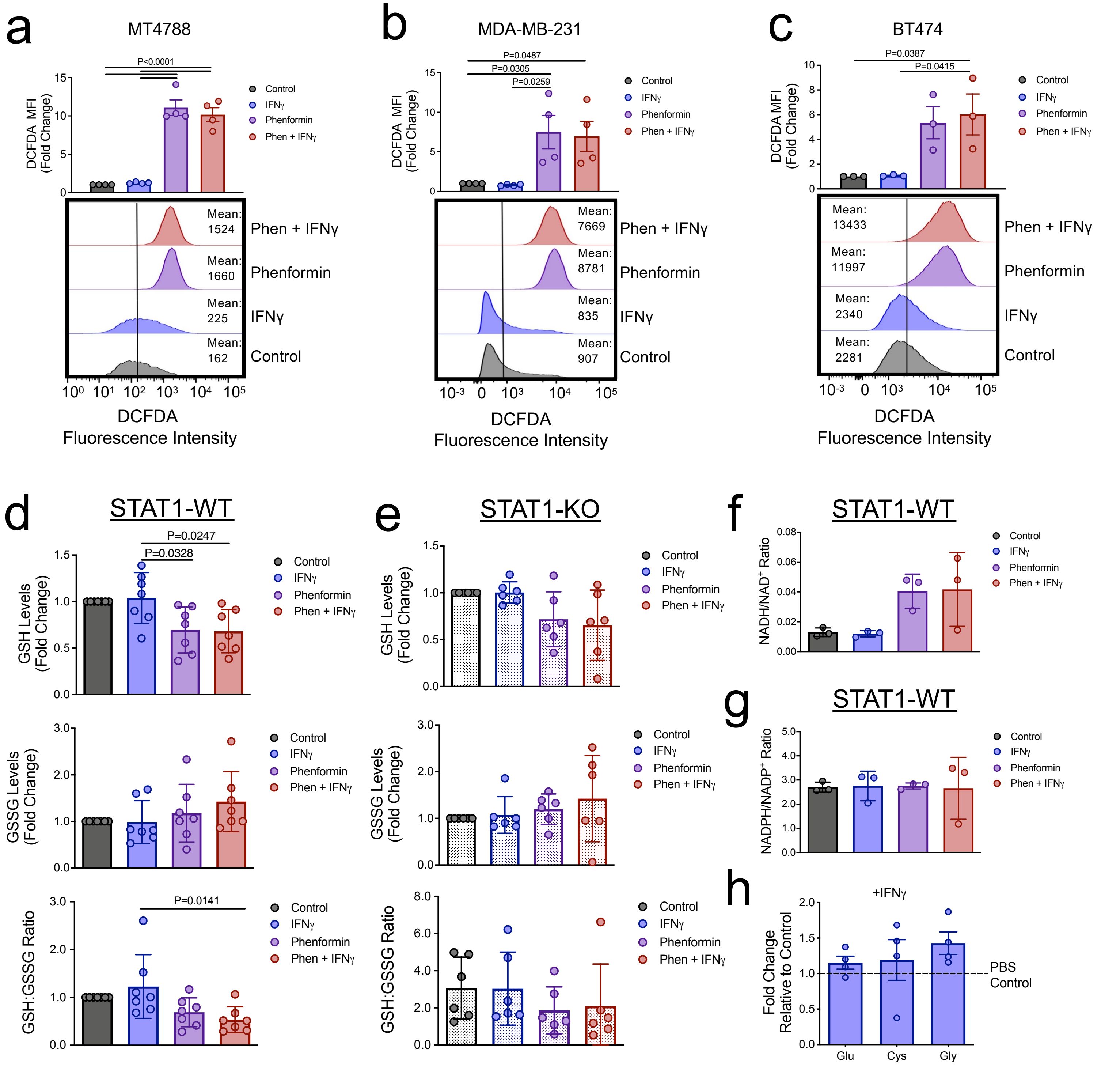

Supplementary Figure 4

Supplementary Figure 4: Phenformin minimally impacts glutathione levels in breast cancer cells.
(a-c) Total ROS levels were measured by DCFDA flow cytometry in **(a)** MT4788, **(b)** MDA-MB-231 and **(c)** BT474 cells treated with IFNγ, phenformin for 24hours. The data represents the average fold change in the DCFDA geometric mean fluorescence intensity (MFI) relative to PBS treated controls (mean of means) ±SEM from **(a, b)** n=4 or **(c)** n=3 independent experiments. Representative histograms are also shown.
(d, e) GSH and GSSG levels, as well as the GSH/GSSG ratio was determined in **(d)** MT4788 WT or **(e)** STAT1-KO cells treated with phenformin and IFNγ alone or in combination, for 36 hours. Data is shown as the average fold change in GSH or GSSG levels or by calculating the GSH/GSSG ratio compared to PBS controls from n=6-7 technical replicates over 2 independent experiments, mean ±SD.
(f, g) Ratios of **(f)** NADH/NAD+ and **(g)** NADPH/NADP+ were determined in MT4788 cells treated with phenformin and/or IFNγ. The data is representative of n=3 independent experiments (mean of means) ±SEM.
(h) Relative glutamic acid, cysteine and glycine levels in MT4788 STAT1-WT and STAT1-KO cells cultured either in the absence or presence of IFNγ for 24 hours. Data is representative of n=4 independent experiments (mean of means) ± SEM.
P values were calculated using a two-way ANOVA with a Tukey's posthoc test.
See also Figure 4.

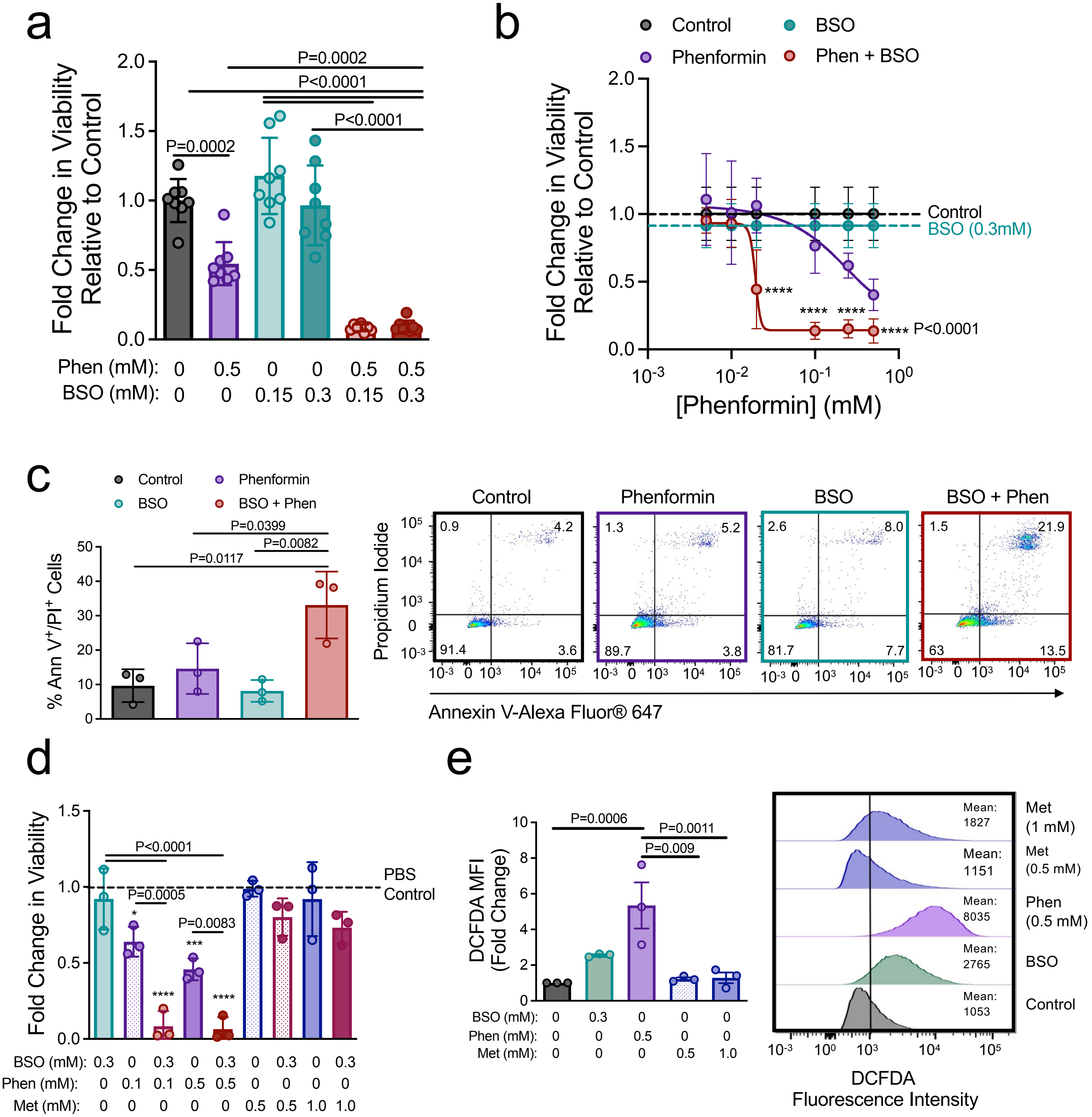

Supplementary Figure 5

Supplementary Figure 5: Phenformin is more potent than metformin in inducing ROS production and in sensitizing breast tumors to BSO treatment.

(a,b) BT474 cells were treated for 48 hours with phenformin, BSO, either alone or in combination, with varying concentrations of **(a)** BSO or **(b)** phenformin as indicated. The data is shown as fold change in viability compared to PBS control mean ±SD and is representative of 2 independent experiments (n=4 technical repeats each). ****P<0.0001.

(c) Percentage of Annexin V+/PI+ cells following treatment with phenformin (500 μM and/or BSO (300 μM) for 40 hours. The data is representative of three independent experiments (±SEM). Representative dot plots are shown.

(d) BT474 cells were treated for 48 hours with BSO and varying concentrations of phenformin or metformin (as indicated), either alone or in combination. The data is shown as fold change in cell viability compared to PBS control and is representative of three independent experiments (mean of means) (±SEM). *P values to Control. *P=0.0154, ***P=0.0006, ****P<0.0001.

(e) Total ROS levels were measured by DCFDA flow cytometry in BT474 cells treated with BSO, phenformin or metformin for 24hours. The data represents the average fold change in the DCFDA geometric mean fluorescence intensity (MFI) relative to PBS treated controls from n=3 independent experiments (mean of means) ±SEM. Representative histograms are also shown. PBS served as the vehicle control.

P values were calculated using a two-way ANOVA with a Tukey's posthoc test.

Other P values are indicated directly in Figure.

See also Figure 5.

a

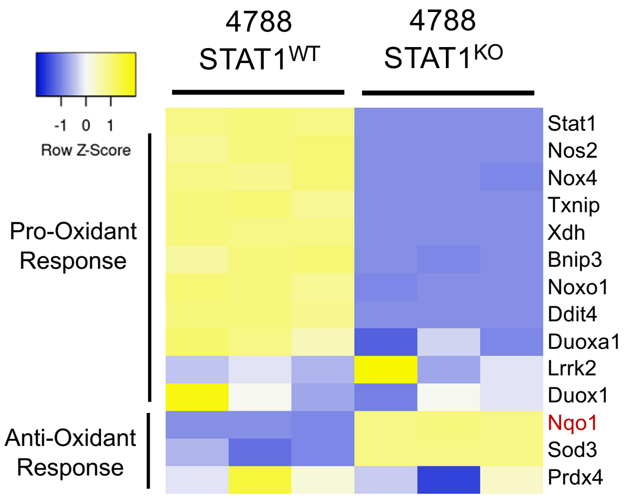

b

GO Term	# Genes	FDR	Fold Enrichment
immune system process	89	4.5E-27	3.667
anti-viral defense	45	1.66E-16	4.252
innate immune response	62	1.34E-10	2.446
antigen processing	20	3.21E-10	5.844
cell migration	36	5.51E-08	2.798
ossification	23	1.03E-07	3.780
IFNγ response	19	1.45E-07	4.409
inflammatory response	48	5.18E-07	2.202
wound healing	20	5.58E-06	3.357
response to hypoxia	30	1.13E-05	2.465
gene expression	49	1.42E-05	1.938
cell adhesion	55	3.84E-05	1.789
regulation of apoptosis	42	3.99E-05	1.978
**ROS regulation**	**11**	**9.21E-05**	**4.567**
cell chemotaxis	16	9.66E-05	3.237
regulation of proliferation	31	1.05E-04	2.155
angiogenesis	32	1.16E-04	2.113
circadian rhythm	19	1.42E-04	2.776
regulation of exocytosis	09	2.50E-04	5.072
ECM organization	19	2.86E-04	2.629
signal transduction	44	4.87E-04	1.736
glucose metabolism	06	7.01E-04	5.049
**superoxide metabolism**	**05**	**0.0289**	**4.152**

c

Gene	864 WT/KO*	864 FDR	4788 WT/KO8	4788 FDR	
STAT1	↑ 34 fold	0	↑ 77 fold	0	
NOS2	↑ 4.8 fold	1.23E-23	↑ 212 fold	8.7E-128	ROS Inducer
NOX4	no change	0.293	↑ 64 fold	4.68E-43	
TXNIP	no change	0.516	↑ 8.6 fold	0	
XDH	↑ 2.4 fold	7.5E-125	↑ 3.6 fold	0	
BNIP3	↑ 1.4 fold	3.74E-07	↑ 3.1 fold	8.7E-110	
NOXO1	↑ 1.2 fold	0.00046	↑ 3.0 fold	1.8E-161	
DDIT4	↓ 1.4 fold	0.0292	↑ 2.6 fold	0	
DUOXA1	↓ 2.1 fold	6.69E-61	↑ 1.3 fold	0.00026	
LRRK2	↑ 3.5 fold	1.95E-33	no change	0.5257	
DUOX1	↓ 3.2 fold	1.1E-97	no change	0.8522	
**NQO1**	**↓ 1.5 fold**	**2.5E-09**	**↓ 3.9 fold**	**0**	ROS Scavenger
SOD3	no change	0.3472	↓ 2.6 fold	2.83E-10	
PRDX4	↑ 2.1 fold	3.9E-102	no change	0.1032	

***Fold change between STAT1WT and STAT1KO cells treated with IFNγ**

Supplementary Figure 6: Nqo1 is identified as a ROS scavenger downstream of the IFNγ-STAT1 axis in MT-transformed breast cancer cells

(a) RNAseq analysis of MT4788-VC and STAT1-KO breast cancer cells stimulated with IFNγ for 24 hours. Heatmaps of the top differentially expressed genes (>2 fold; FDR <0.05) controlling known IFNγ-regulated pathways.

(b) Gene Ontology (GO) terms that are most differentially expressed between IFNγ-treated STAT1-WT and STAT1-KO 4788 breast cancer cells as determined by RNA sequencing. The number of differentially expressed genes, false discovery rate (FDR) and fold enrichment for each GO term are shown. A complete list of all GO terms, including the gene lists assigned in each category can be found in Supplemental Data File 3, where P values were derived from Gene Set Enrichment Analysis.

(c) RNA seq data showing differentially regulated genes involved in redox control, including ROS inducers and ROS scavengers. The fold change in gene expression (WT/KO) and false discovery rate (FDR) for each gene is shown across both cell lines. The data is representative of n=3 biological repeats per condition.

P values in Supplemental data files 1 and 2 were calculated by DESeq2 using a Wald test corrected by the Benjamini and Hochberg method.

See also Figure 6 and Supplementary Data Files 1, 2, 3.

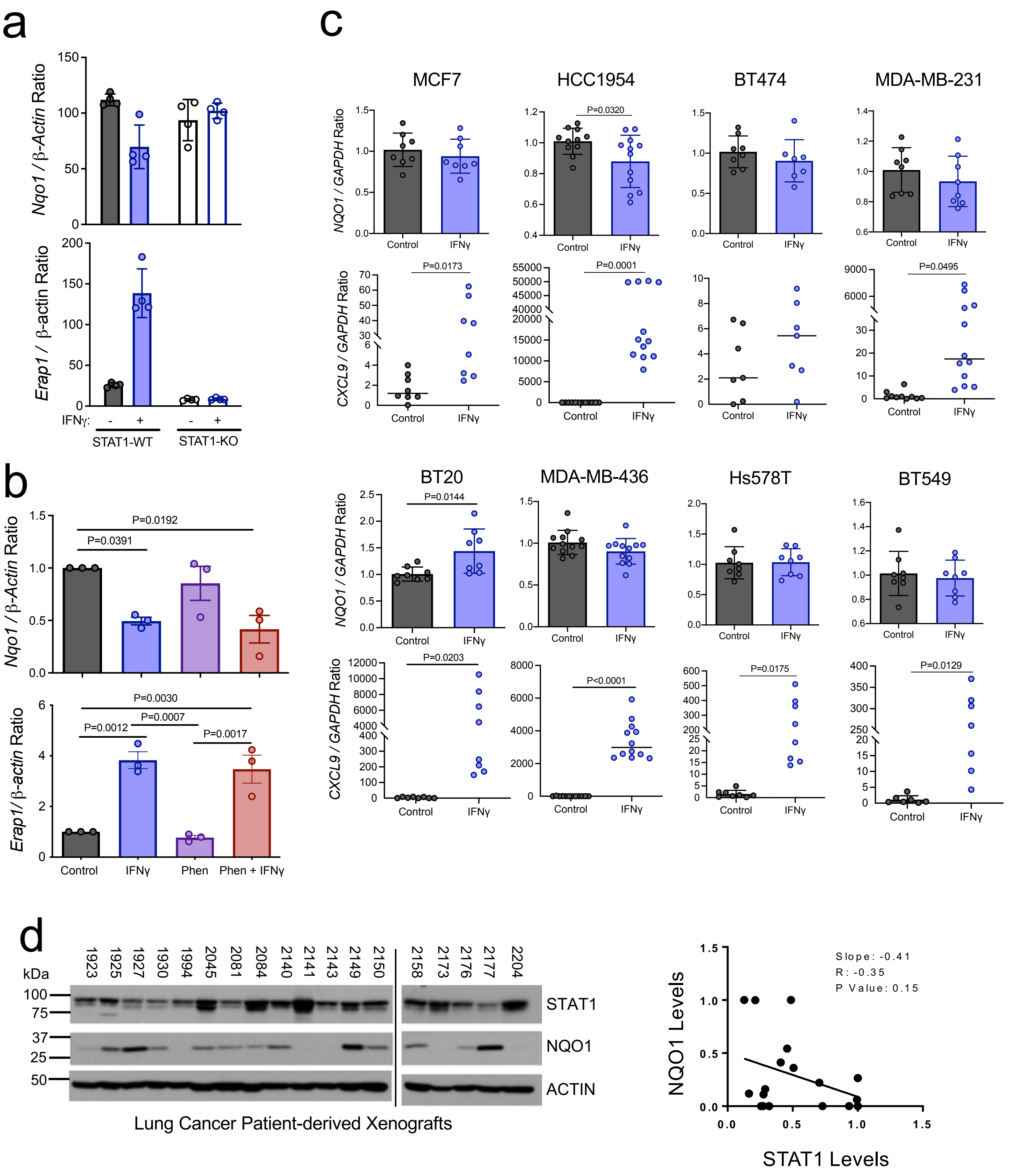

Supplementary Figure 7

Supplementary Figure 7: Nqo1 levels are inversely correlated with STAT1 levels or STAT1 transcriptional activity in lung and breast cancers.
(a) RT-qPCR analysis in control and IFNγ-stimulated (24h) STAT1-WT and STAT KO cells (n=4 technical repeats; mean ±SD for each condition), expressed as a ratio to ACTIN.
(b) RT-qPCR analysis in control, IFNγ, phenformin, cotreated or single treated (24h) MT4788 cells (n=3 independent experiments (mean of means) (±SEM).
(c) RT-qPCR analysis in control and IFNγ-stimulated (24h) from human cell lines from Figure 1c and 6b, c, (MCF7: n=8; HCC1954: Control: n=11, IFNγ: N=12; BT474: Control: n=8, IFNγ: N=7; MDA-MB-231: n=8; BT20: n=8; MDA-MB-436: n=12; Hs578t: n=8; BT549: n=8; technical repeats over 2 independent experiments each; mean ±SD) expressed as a ratio to GAPDH.
(d) Immunoblot analysis of tumor lysates from 18 independent lung cancer brain metastasis patient-derived xenografts, using STAT1, NQO1 and β-Actin specific antibodies. Densitometric quantification of the immunoblots shown, represented as relative NQO1 and STAT1 levels (normalized to β-Actin levels) was performed by Image J software.
P values were calculated using a one-way ANOVA with a Tukey's posthoc test (b) and two-tailed unpaired t-tests (panel c). A Pearson's correlation (linear regression analysis) was used to calculate the relationship between NQO1 and STAT1 levels (panel d).
See also Figure 6.

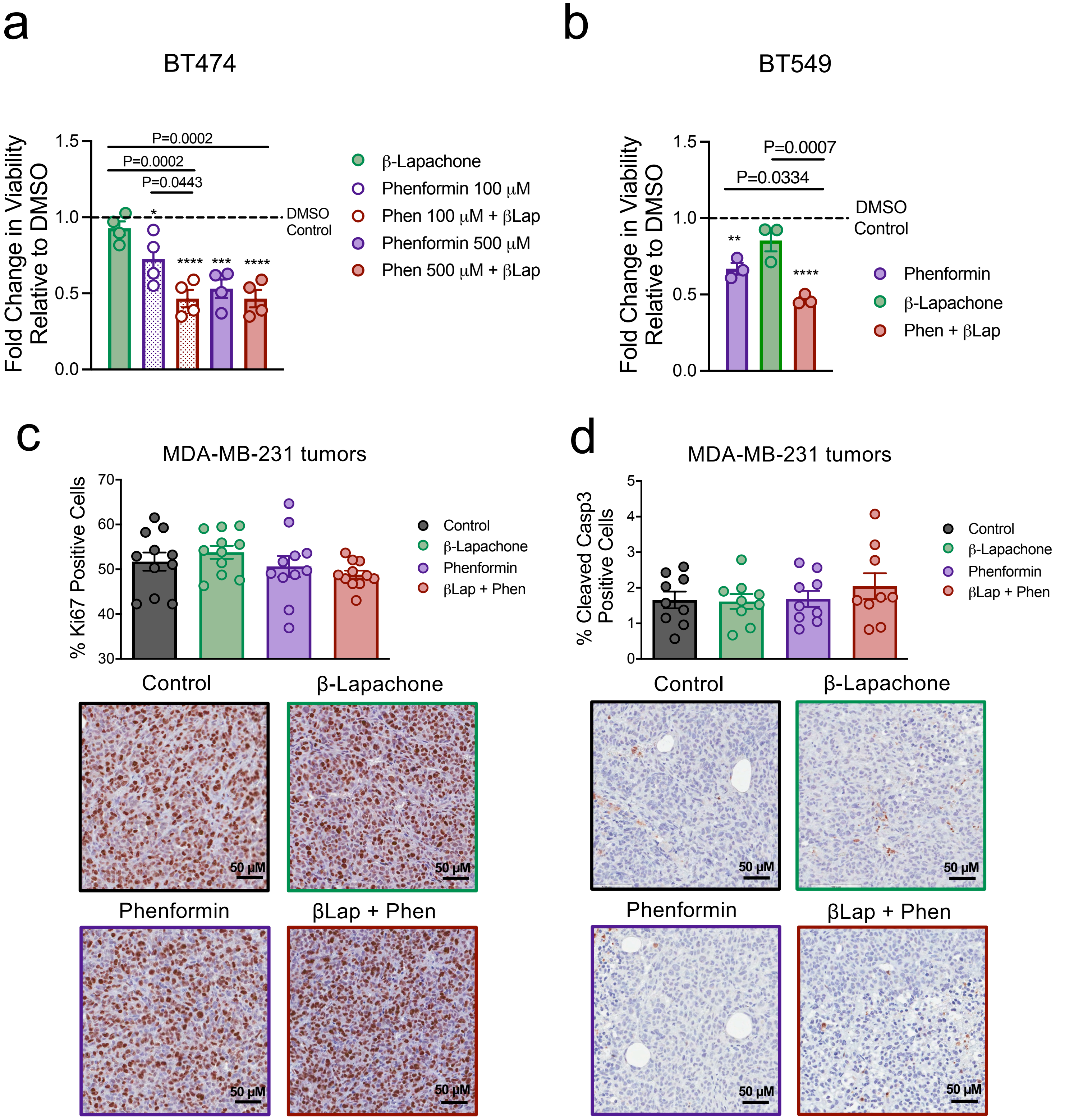

Supplementary Figure 8

Supplementary Figure 8: Breast cancer cells are sensitive to the anti-tumorigenic effects of β-lapachone/phenformin combination treatment.

(a, b) Viability of **(a)** BT474 cells treated with phenformin (0.1 or 0.5 mM), β-lapachone (1 μM); and *P=0.0307, ***P=0.0002 ****P<0.0001 **(b)** BT549 cells, treated with phenformin (0.1 mM), β-lapachone (0.5 μM); alone or in combination, for 48 hours. ** P=0.0020, ****P<0.0001. Data is shown as the fold change in cell viability relative to DMSO control, n=3 independent experiments (mean of means) ±SEM.

(c, d) Immunohistochemical analysis of mammary tumors described in Figure 7d using **(c)** Ki67 n=11 tumors/group and **(d)** cleaved caspase-3-specific antibodies n=9 tumors/group. The data is shown as the mean % positive cells ±SEM. Representative images are shown.

P values were calculated using a two-way ANOVA with a Tukey's posthoc test. *P=0.0307, ***P*<0.01, ****P*<0.001, *****P*<0.0001. *P values indicate comparison to DMSO treatment, other P values are indicated in Figure.

See also Figure 7.

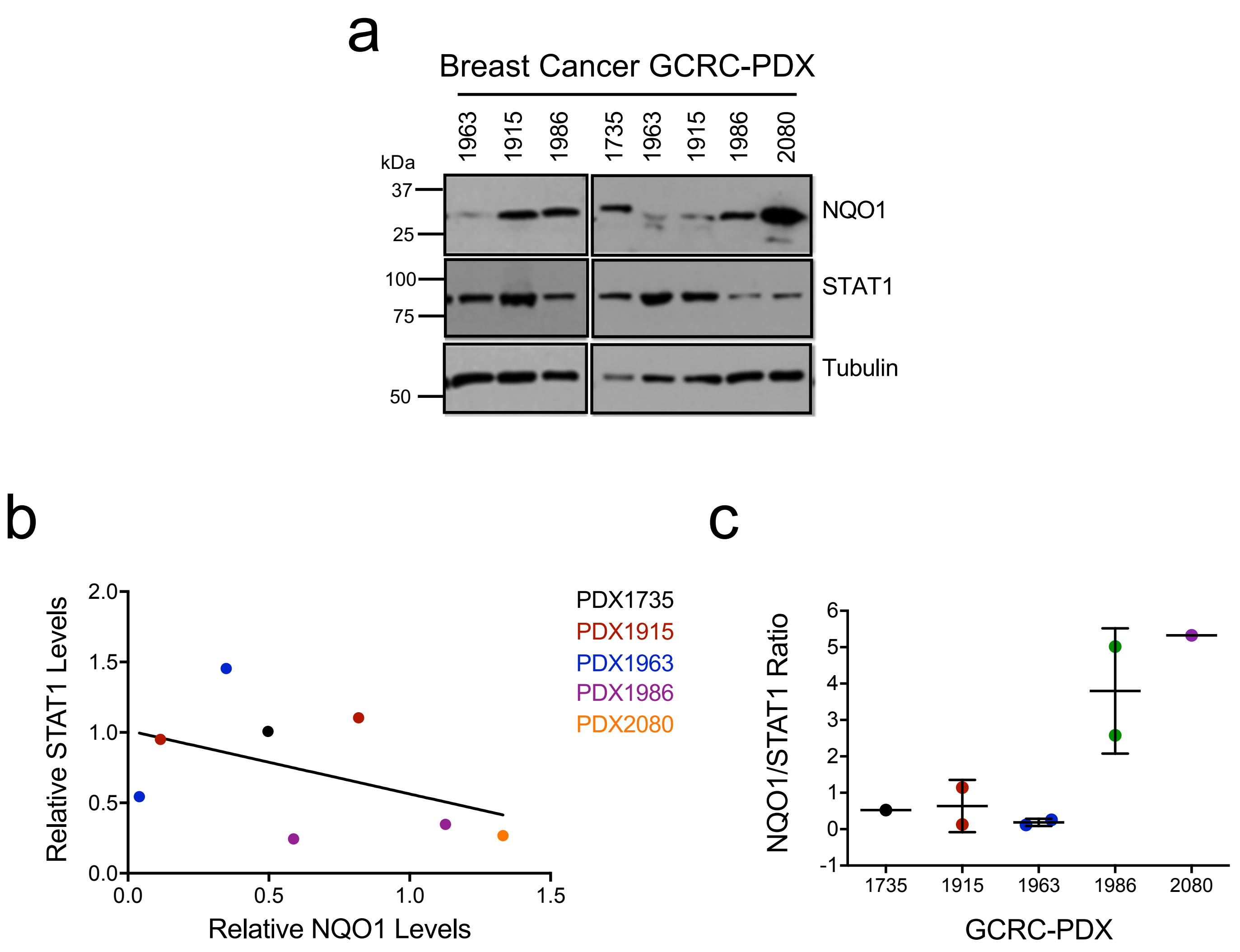

Supplementary Figure 9

Supplementary Figure 9: Nqo1 and STAT1 levels are inversely correlated in triple negative breast cancer patient derived xenografts.

(a) Immunoblot analysis of cell lysates from five independent breast tumor PDXs using NQO1-, STAT1- and Tubulin-specific antibodies. The immunoblots shown in panel A were quantified by ImageJ software and **(b)** relative expression levels (normalized to Tubulin levels) were either plotted individually or **(c)** the NQO1/STAT1 ratio for individual PDXs was also determined, 1735, 2080: n=1 sample; 1915, 1963, 1986 n=2 biological repeats, mean ±SD.

See also Figure 8.

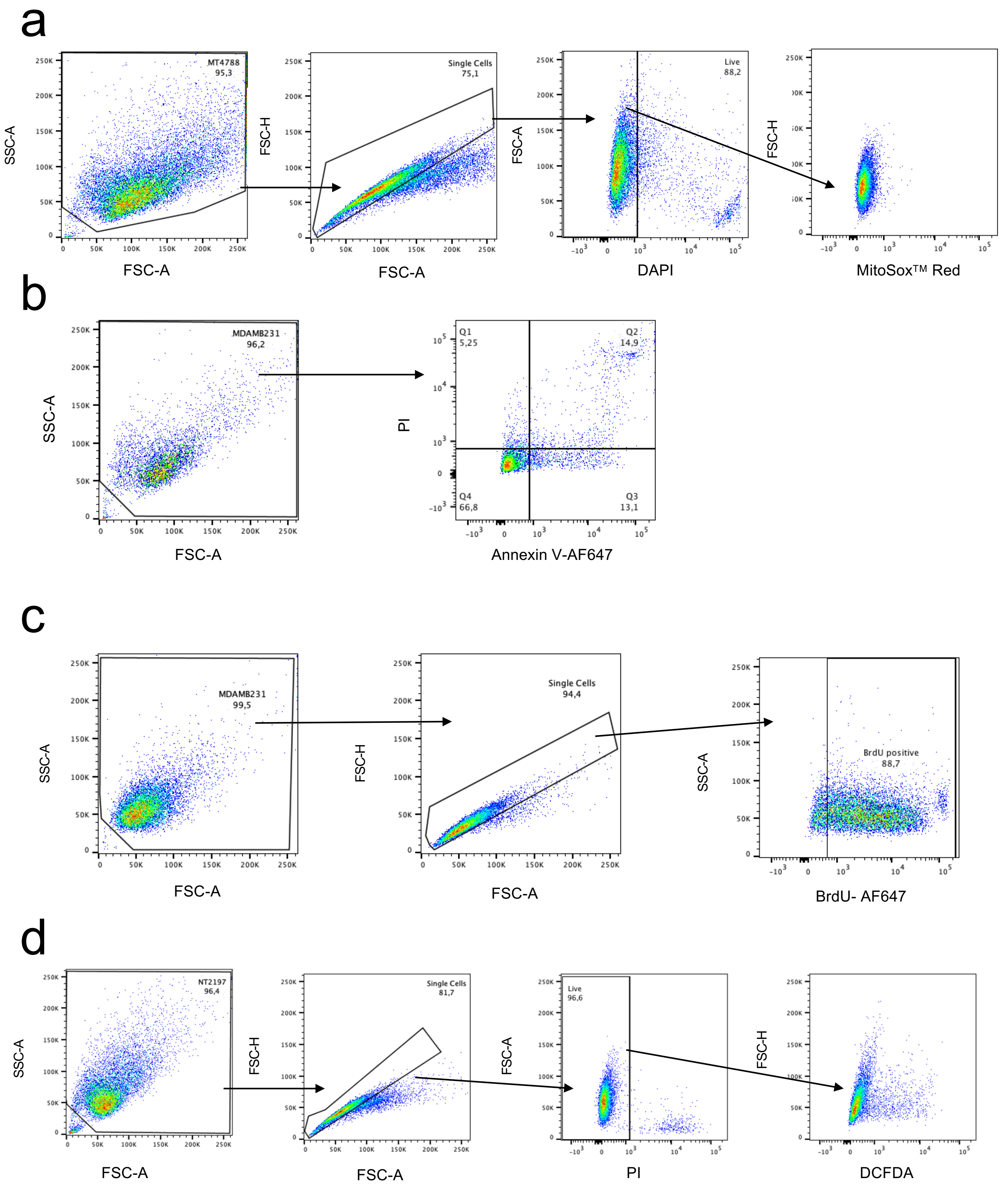

Supplementary Figure 10

Supplementary Figure 10: Gating strategies for flow cytometry experiments
(a) Gating strategy for *MitoSOX^{TM} Red Mitochondrial superoxide indicator* experiments (Figures 4a, 4b)
(b) Gating strategy for Annexin V and PI experiments (Figures 5d, 7b, S5c).
(c) Gating strategy for BrdU incorporation assay gating was performed (Figures 5e, 7c).
(**d**) Gating strategy for DCFDA experiments (Figures 8a, 8d, S4a, S4b, S4c, S5e).

Supplementary Table 1: Antibody information used for immunohistochemical staining of paraffin-embedded sections

Antibody	Company	Identifier	Dilution	Antigen retrieval buffer
STAT1 p84/p91 (E-23)	Santa Cruz	Sc346	1:750	Citrate
Ki67	Abcam	ab15580	1:500	Citrate
Cleaved Caspase-3 (Asp175)	Cell Signaling	9661	1:250	Citrate
8 oxo-dG[1]	Trevigen	4354-MC-050	1:2000	No retrieval
phospho-AMPK α (Thr172)	Cell Signaling	2535	1:100	Citrate
Anti-Granzyme B	Abcam	ab4059	1:300	Citrate
Biotinylated anti-Rabbit (Horse)	Vector Laboratories	BA-1100	1:1000	N/A

[1] Mouse on Mouse polymer kit used instead of secondary anti-Rabbit.

Supplementary Table 2: Antibody conditions used for immunoblot analysis

Antibody	Company	Identifier	Dilution
Anti-STAT1 (D4Y6Z)	New England Biolabs	14995S	1:1000
Anti-phosphoY701- STAT1 (58D6)	New England Biolabs	9167S	1:1000
Anti-Tubulin	Sigma	T5168	1:10000
Anti-Actin (H-6)	Santa Cruz Biotechnology	sc-376421	1:10000
Anti-NQO1 for human (clone: A180) (anti-mouse)	Santa Cruz Biotechnology	sc-32793	1:500
WB:Anti-NQO1 (for Mouse)	Abcam	ab34173	1:1000

Supplementary Table 3: Nucleotide sequences of primers used for RT-qPCR analysis and or shRNAs

Application	Gene	Species	Sequence	Identification number
RT-PCR	*β-ACTIN*	Human	Forward: TGCTATCCCTGTACGCCTCT<br>Reverse: TAATGTCACGCACGATTTCC	
RT-PCR	*IRF7*	Human	Forward: TACCATCTACCTGGGCTTCG<br>Reverse: AGGGTTCCAGCTTCACCA	
RT-PCR	*NQO1*	Human	Forward: GGGATCCACGGGGACATGAATG<br>Reverse: ATTTGAATTCGGGCGTCTGCTG	
RT-PCR	*STAT1*	Human	Forward: CGGCTGAATTTCGGCACCT<br>Reverse: CAGTAACGATGAGAGGACCCT	
RT-PCR	*β-Actin*	Mouse	Forward: GGCTGTATTCCCCTCCATCG<br>Reverse: CCAGTTGGTAACAATGCCATGT	
RT-PCR	*Erap1*	Mouse	Forward: TAATGGAGACTCATTCCCTTGGA<br>Reverse: AAAGTCAGAGTGCTGAGGTTTG	
RT-PCR	*Gapdh*	Mouse	Forward: AACGACCCCTTCATTGAC<br>Reverse: TCCACGACATACTCAGCAC	
RT-PCR	*Irf9*	Mouse	Forward: GCCGAGTGGTGGGTAAGAC<br>Reverse: GCCGAGTGGTGGGTAAGAC	
RT-PCR	*Nqo1*	Mouse	Forward: TTCTGTGGCTTCCAGGTCTT<br>Reverse: AGGCTGCTTGGAGCAAAATA	
RT-PCR	*Psmb8*	Mouse	Forward: GTGCAGGTTGTATTATCTTCGGA<br>Reverse: CGAGTCCCATTGTCATCTACG	
shRNA	*Nqo1*	Mouse	(Gene ID: 18104) 1: CCGGCCATCAAGATTCGTTGTCTATCTCGAGATAGACAACGAATCTTGATGGTTTTTG	TRCN0000041863
shRNA	*Nqo1*	Mouse	(Gene ID: 18104) 2: CCGGCCGAGTCATCTCTAGCATATACTCGAGTATATGCTAGAGATGACTCGGTTTTTG	TRCN0000041864
shRNA	*Nqo1*	Mouse	(Gene ID: 18104):3 CCGGCCCATTCAGAGAAGACATCATCTCGAGATGATGTCTTCTCTGAATGGGTTTTTG	TRCN0000041867
shRNA	*NQO1*	Human	(Gene ID:18104) 1: CCGGTGGAAGAAACGCCTGGAGAATCTCGAGATTCTCCAGGCGTTTCTTCCATTTTTG	TRCN0000350361
shRNA	*NQO1*	Human	Gene ID:18104) 2: CCGGTGGAAGAAACGCCTGGAGAATCTCGAGATTCTCCAGGCGTTTCTTCCATTTTT	TRCN0000003768
shRNA	*NQO1*	Human	(Gene ID:18104) 3: CCGGAGACCTTGTGATATTCCAGTTCTCGAGAACTGGAATATCACAAGGTCTTTTTT	TRCN0000003769
shRNA	*NQO1*	Human	(Gene ID:18104) 4:	TRCN0000003766

Supplementary Table 3 continued: Nucleotide sequences of primers used for RT-qPCR analysis and or shRNAs

			CCGGAGAAAGGACATCACAGGTAAACTCGAGTTTACCTGTGATGTCCTTTCTTTTTT	
shRNA	*NQO1*	Human	(Gene ID:18104) 5: CCGGCATGTTATCAAATCTGGGTATCTCGAGATACCCAGATTTGATAACATGTTTTT	TRCN0000003770
shRNA		Non-mammalian	MISSION® PLKO.1-Puro Non-Mammalian shRNA control plasmid DNA	SHC002
shRNA		Non-mammalian	MISSION®PLKO.5-Puro Non-mammalian shRNA control plasmid DNA	SHC202

shRNAs were obtained from Genetic Perturbation Service at McGill University and Dr. Sidong Huang
MISSION® shRNA library also available through Sigma-Aldrich.
