# Supplementary material for: STAT1 potentiates oxidative stress revealing a targetable vulnerability that increases phenformin efficacy in breast cancer
Source: Nat Commun. 2021 Jun 3;12:3299. doi: 10.1038/s41467-021-23396-2 (PMC8175605; doi:10.1038/s41467-021-23396-2)
Supplement: Supplementary file 3 — Description of Additional Supplementary Files [file 41467_2021_23396_MOESM3_ESM.pdf]

Description of Additional Supplementary Files

File name: Supplementary Data 1
Description: RNAseq analysis of MT864-VC and STAT1-KO breast cancer cells stimulated with IFNγ for 24 hours. P values in were calculated by DESeq2 using a Wald test corrected by the Benjamini and Hochberg method.

File name: Supplementary Data 2
Description: RNAseq analysis of MT4788-VC and STAT1-KO breast cancer cells stimulated with IFNγ for 24 hours. P values in were calculated by DESeq2 using a Wald test corrected by the Benjamini and Hochberg method.

File name: Supplementary Data 3
Description: Complete list of Gene Ontology (GO) terms that are most differentially expressed between IFNγ-treated STAT1-WT and STAT1-KO 4788 breast cancer cells as determined by RNA sequencing, where P values were derived from Gene Set Enrichment Analysis.
