# Supplementary material for: STAT1 potentiates oxidative stress revealing a targetable vulnerability that increases phenformin efficacy in breast cancer
Source: Nat Commun. 2021 Jun 3;12:3299. doi: 10.1038/s41467-021-23396-2 (PMC8175605; doi:10.1038/s41467-021-23396-2)
Supplement: Supplementary file 7 — Reporting summary [file 41467_2021_23396_MOESM7_ESM.pdf]

nature research

Corresponding author(s): Josie Ursini-Siegel

Last updated by author(s): Apr 6, 2021

Reporting Summary

Nature Research wishes to improve the reproducibility of the work that we publish. This form provides structure for consistency and transparency in reporting. For further information on Nature Research policies, see our Editorial Policies and the Editorial Policy Checklist.

Statistics

For all statistical analyses, confirm that the following items are present in the figure legend, table legend, main text, or Methods section.

n/a	Confirmed	
☐	☒	The exact sample size (*n*) for each experimental group/condition, given as a discrete number and unit of measurement
☐	☒	A statement on whether measurements were taken from distinct samples or whether the same sample was measured repeatedly
☐	☒	The statistical test(s) used AND whether they are one- or two-sided<br>*Only common tests should be described solely by name; describe more complex techniques in the Methods section.*
☐	☒	A description of all covariates tested
☐	☒	A description of any assumptions or corrections, such as tests of normality and adjustment for multiple comparisons
☐	☒	A full description of the statistical parameters including central tendency (e.g. means) or other basic estimates (e.g. regression coefficient) AND variation (e.g. standard deviation) or associated estimates of uncertainty (e.g. confidence intervals)
☐	☒	For null hypothesis testing, the test statistic (e.g. *F*, *t*, *r*) with confidence intervals, effect sizes, degrees of freedom and *P* value noted<br>*Give P values as exact values whenever suitable.*
☒	☐	For Bayesian analysis, information on the choice of priors and Markov chain Monte Carlo settings
☐	☒	For hierarchical and complex designs, identification of the appropriate level for tests and full reporting of outcomes
☐	☒	Estimates of effect sizes (e.g. Cohen's *d*, Pearson's *r*), indicating how they were calculated

Our web collection on statistics for biologists contains articles on many of the points above.

Software and code

Policy information about availability of computer code

Data collection	BD FACSDiva TM v8.0.1 Software; Agilent Seahorse Wave Controller Software 2.4; LC/MS Data Acquisition for 6400 series triple quadrupole Version 10.0 build 10.0.127, Agilent GC/MS data collection software, ChemStation E01.02.1177.
Data analysis	Graphpad Prism v6 and v9; Aperio Imagescope v12; FlowJo 10 Software; Trimmomatic v0.32; STAR v2.3.0e; DESeq2 v1.14.1; featureCounts v1.4.4; FASTQC v0.11.2; BEDtools v2.17.0; MassHunter Quantitative Analysis Software for MSD and QQQ,Version 10.2 build 10.2.733.8; Microsoft Excel v16.46.

For manuscripts utilizing custom algorithms or software that are central to the research but not yet described in published literature, software must be made available to editors and reviewers. We strongly encourage code deposition in a community repository (e.g. GitHub). See the Nature Research guidelines for submitting code & software for further information.

Data

Policy information about availability of data

All manuscripts must include a data availability statement. This statement should provide the following information, where applicable:

- Accession codes, unique identifiers, or web links for publicly available datasets
- A list of figures that have associated raw data
- A description of any restrictions on data availability

RNA sequencing data that support the findings of this study have been deposited in Gene Expression Omnibus with the accession code GSE153189, https://www.ncbi.nlm.nih.gov/geo/query/acc.cgi?acc=GSE153189. Mouse reference genome mm10 used in this study can be accessed here: http://ccb.jhu.edu/software/tophat/igenomes.shtml (under Mus Musculus/UCSC/mm10). Uncropped versions of scans of western blots can be found in the Source Data section. Supplementary Data files 1, 2 and 3 have been provided for the RNAseq analyses presented in Figure 6a and Supplementary Figure S6a-c. Further information and requests for resources and reagents should be directed to and will be fulfilled by the corresponding author, Josie Ursini-Siegel (giuseppina.ursini-siegel@mcgill.ca).

Field-specific reporting

Please select the one below that is the best fit for your research. If you are not sure, read the appropriate sections before making your selection.

[x] Life sciences [ ] Behavioural & social sciences [ ] Ecological, evolutionary & environmental sciences

For a reference copy of the document with all sections, see nature.com/documents/nr-reporting-summary-flat.pdf

Life sciences study design

All studies must disclose on these points even when the disclosure is negative.

Sample size	For the in vivo studies, power calculations were performed assuming a two fold change between control and experimental groups with a standard deviation of 70%, a type I error rate of 0.05 and 0.8 for the desired power. This required a minimum of 8 tumors per experimental group. Note that the observed differences in this study were within or exceeded the assumed differences when we initially established our sample size calculations.<br><br>For in vitro studies, sample size of 3-6 independent experiments were performed. Exception to this was Figure 1 e with 2 independent repeats each with 4 technical samples, in two cell line systems. No sample size calculation was performed before hand. Exact sample sizes are defined in the figure legends.
Data exclusions	1 biological repeat of the seahorse experiments was excluded (Figure S3) due to the total cell number less than 1/2 of the other 4 independent experiments, as determined from a parallel plate after analyzing the experiment. Confluency of cells is known to impact results of this assay and resulted in outlier values.
Replication	All attempts to reproduce data were successful. Number of independent repeats are described in figure legends to ensure reproducibility of results.
Randomization	Mice were randomized to treatment groups prior to the start of experiments. For in vitro studies, within an independent experiment, all treated cells were plated from the same tissue culture plate of cells. For biological repeats, new vials of cells were thawed and performed on separately. Treatment groups were divided randomly and equally. Sample collection was done in a random order. For Seahorse experiments, particular attention was made to ensure that independent repeats had a different plate layout, in terms of treatments, to account for any instrument variability. For GC/MS, LC/MS, flow cytometry, samples were loaded and analyzed in a random order.
Blinding	Blinding was not performed for mouse experiments because of daily treatment of mice. Due to obvious differences between treatment groups, most in vitro assays were not blinded. The same results have been repeated by multiple members of the research team. For flow cytometry, qPCR and RNA seq experiment, researchers were not blinded. Blinding was performed for immunohistochemistry staining. The LC/MS data collection and analysis was performed by the core facility members. The GC/MS data collection were performed by the core facility members and researchers, the analysis was performed by two research members.

Reporting for specific materials, systems and methods

We require information from authors about some types of materials, experimental systems and methods used in many studies. Here, indicate whether each material, system or method listed is relevant to your study. If you are not sure if a list item applies to your research, read the appropriate section before selecting a response.

Materials & experimental systems

n/a	Involved in the study	
[ ]	[x]	Antibodies
[ ]	[x]	Eukaryotic cell lines
[x]	[ ]	Palaeontology and archaeology
[ ]	[x]	Animals and other organisms
[ ]	[x]	Human research participants
[x]	[ ]	Clinical data
[x]	[ ]	Dual use research of concern

Methods

n/a	Involved in the study	
[x]	[ ]	ChIP-seq
[ ]	[x]	Flow cytometry
[x]	[ ]	MRI-based neuroimaging

Antibodies

Antibodies used	IHC antibodies:<br>STAT1 p84/p91 (E-23) Santa Cruz, Sc346, 1:750 [replaced by Anti-Stat1 Antibody (C-136): sc-464];<br>Ki67 Abcam ab15580 1:500;<br>Cleaved Caspase-3 (Asp175) Cell Signaling, 9661, 1:250;<br>8 oxo-dG1 Trevigen (15A3) 4354-MC-050 1:2000;<br>phospho-AMPK α (Thr172) Cell Signaling 2535 1:100;

Anti-Granzyme B Abcam ab4059 1:300;
Biotinylated anti-Rabbit (Horse) Vector Laboratories BA-1100 1:1000.

Immunobloting:
Anti-STAT1 (D4Y6Z) New England Biolabs 14995S 1:1000;
Anti-phosphoY701- STAT1 (58D6) New England Biolabs 9167S 1:1000;
Anti-Tubulin Sigma T5168 1:10000;
Anti-Actin (H-6) Santa Cruz Biotechnology sc-376421 1:10000;
Anti-NQO1 for human (clone: A180) (anti-mouse) Santa Cruz Biotechnology sc-32793 1:500;
Anti-NQO1 (for Mouse) Abcam ab34173 1:1000.

Flow Cytometry:
Anti-BrdU, clone 3D4, Phase flow Kit- Biolegend, 370706;
Annexin V Alexa Fluor 647 (Biolegend, 640912)

Anti-PD1 in vivo treatment:
Mice were treated every 3 days with 100μg of either the anti-PD1 antibody (InVivoMAb, clone RMP1-14, BioXCell, cat # BP0146) or isotype control IgG (InVivoMAb Rat IgG2a, clone 2A3, BioXCell, cat # BE0089).

Validation

Antibodies were validated by the commercial supplier. In addition to manufacturer validation described below, for flow cytometry we included corresponding negative controls (no BrdU incubation), positive (no treatment and heat-induced cell death for Annexin V/PI). Additionally, for all IHC experiments we use secondary ab only controls, positive controls and when available, additional negative controls such as (ie. STAT1-/- tumors for STAT1 antibody validation).

The following validations were taken from manufacturer websites: Anti-PD1 (RMP1-14): was validated by the manufacturer and shown by "Western blot to detect purified reduced mouse PD-1", dependent on amount of PD1 loaded. Isotype control: 2A3 was ideal as an isotype matched control because it detects "trinitrophenol which is not expressed by mammals". Annexin V-AF647 was validated by manufacturer as follows: "Jurkat cells were induced with 10 μM camptothecin, cells were washed and stained with Annexin V-Alexa Fluor® 647 conjugate, and SYTOX® Green nucleic acid stain. Cells were analyzed from flow cytometry using 488 and 633 nm excitation." Anti-NQO1 ab34173 was shown by manufacturers by Western blot to "specifically react with NQO1 in wild-type HAP1 cells as signal was lost in NQO1 knockout cells". NQO1 Antibody (A180): sc-32793. was validated by "near-infrared western blot analysis of NQO1 expression in non-transfected: sc-117752 (A) and human NQO1 transfected: sc-172015 (B) whole cell lysates." They also showed that the NQO1 Antibody (A180) detected 31kDa band in HepG2, SW480, HCT 116 whole cell lysates. Actin (H-6): sc-376421 was validated by "Western blot analysis of Actin expression in HeLa, NIH/3T3 and KNRK whole cell lysates." Anti-Tubulin Sigma T5168 was validated by "Enhanced Validation-By Independent Antibodies where Chicken fibroblasts cells were fixed and permeabilized with methanol followed by acetone. Fixed cells were stained with Monoclonal Anti-α-Tubulin antibody produced in Mouse, Clone: DM1A (Cat. No. T9026). The antibody was developed using 1:40 Goat Anti-Mouse IgG (Fab specific)-FITC antibody (Cat. No. F5262), and 1 μg/mL Monoclonal Anti-α-Tubulin antibody produced in Mouse, Clone: B-5-1-2 (Cat. No. T5168).
Two Anti-α-Tubulin antibodies, T9026 (A) and T5168 (B) target different regions of α-Tubulin show similar staining profiles between the two antibodies, demonstrating Independent Antibody Verification." Anti-phosphoY701- STAT1 (58D6) New England Biolabs 9167S was validated by "Western blot analysis of extracts from HeLa cells untreated or treated with interferon-α (IFN-α), using Phospho-Stat1 (Tyr701) (58D6) Rabbit mAb." Anti-Granzyme B Abcam ab4059: "Synthetic peptide within Human Granzyme B aa 1-100" and validated by IHC to only "stain Granzyme B-positive cytotoxic cells in a GBM xenograft with rejection model". Phospho-AMPK α (Thr172) (Cell Signaling 2535) was validated by "Western blot analysis of extracts from C2C12 cells, untreated or oligomycin-treated (0.5 μM), using Phospho-AMPKα (Thr172) (40H9) Rabbit mAb ". 8 oxo-dG1 Trevigen (15A3) 4354-MC-050 was validated by manufacturer by Immunocytochemistry comparing staining "H2O2 treated and untreated MCF-10A cells using an Alexa Fluor 488 conjugated anti-mouse secondary antibody". Cleaved Caspase-3 (Asp175) Cell Signaling, 9661 was validated by "Immunohistochemical analysis of paraffin-embedded mouse embryo, using Cleaved Caspase-3 (Asp175) Antibody preincubated with control peptide (left) or Cleaved Caspase-3 (Asp175) Blocking Peptide #1050". Ki67 Abcam ab15580 was developed against "Synthetic peptide corresponding to Human Ki67 aa 1200-1300 (internal sequence) conjugated to keyhole limpet haemocyanin." and was shown to stain the nuclei of proliferating cell, by immunohistochemistry (on PFA-fixed paraffin embedded samples" and immunofluerescence. They show a "decrease in Ki67 expression correlating with increased concentration of NADA (N-Arachidonyldopamine". In addition to our own validation, STAT1 p84/p91 (E-23) Santa Cruz, Sc346 "epitope mapping near the C-terminus of Stat1 p84/p91 of human origin, validated by "IIndependent antibody verification showing anti-Stat1/p91/p84 (E-23) and anti-Stat1 p91 (C-24) western blots anlalysis of whole cell lysates from HeLa (medium Stat1 expression), A-431 (high STAT1) and K-562 cells (low Stat1 expression); with both Stat1alpha p91 and Stat1 beta p84 bands detected for anti-Stat1 (E-23)."

Eukaryotic cell lines

Policy information about cell lines

Cell line source(s)

Human: From ATCC.
MDA-MB-231, ATCC HTB-26
BT474, ATCC HTB-20
BT549, ATCC HTB-122
BT20, ATCC HTB-19
MDA-MB-436, HTB-130

	HCC1954, ATCC CRL-2338,<br>Hs578T, ATCC HTB-126.<br><br>Mouse:<br><br>MT4788, MT864 generated in our laboratory and their STAT1-/- counterparts and VC counterparts.<br>NOP6, NOP23 are mammary cell lines that were generated from separate transgenic mice that overexpress Her2/neu OT-I/OT-II under the MMTV promoter, and kindly provided by Dr. Brad Nelson. DOI: 10.1158/0008-5472.CAN-07-0622<br>NMuMG parental and ErbB2-transformed murine counterparts, NT2197 (PMID: 18273058), ATCC CRL-1636<br>4T1-537 (PMID: 20711474). Lung metastatic variant derived from parental 4T1 (Parental line: ATCC CRL-2539)<br><br>Cell lines from Patient-Derived Xenografts:<br>GCRC2080, GCRC1735, GCRC1971, GCRC1986 and GCRC1963 (Dr. Morag Park) (DOI: 10.1038/s42003-020-1042-x)<br>PDX CRC-132 (DOI: 10.1038/s41467-019-10138-8). (JGH breast biobank (protocol # 05-006)).<br>PDX lung cancer brain metastases : 1923, 1925, 1927, 1930, 1994, 2045, 2081, 2084, 2140, 2141, 2143, 2149, 2150, 2158, 2173, 2176, 2177, 2204 (Dr. Peter Siegel and Dr. Kevin Petrecca) (DOI: 10.1093/neuonc/noab002)
Authentication	We have not authenticated the cell lines used in this study.
Mycoplasma contamination	Cells used for experiments were mycoplasma negative.<br>Mycoplasma testing was performed at least once per month, and minimally 24 hours prior any in vivo experiment.
Commonly misidentified lines<br>(See ICLAC register)	No misidentified cell lines were used in this study, to the best of our knowledge.

Animals and other organisms

Policy information about studies involving animals; ARRIVE guidelines recommended for reporting animal research

Laboratory animals	Strains: FVB, SCID-beige, BALB/c, IFNγ-/- and CD8-/- female mice (Charles River Laboratories); Female IFNγ-/- and CD8-/- were already backcrossed onto an FVB background as previously (PMID: 28276425). Female NSG mice were used for patient-derived xenografts (The Jackson Laboratories, Strain # 005557).<br>Age: mammary fat pad injection studies between 7-10 weeks old. Age matched within an experiment and between groups.<br><br>All mice had ad libitum access to food and water and housed within the animal facilities of the Lady Davis Institute.<br>Enrichment cages with cardboard huts, nesting material (NESTLET), solid bottom with corn cob bedding. 2-5 mice per cage.<br>12h light day cycle, mean temperature 22.5 degrees C ± 1.5 degrees C. Humidity: 22-28%.
Wild animals	We did not use any wild animals in this study.
Field-collected samples	No samples were collected from the field.
Ethics oversight	These studies were approved by and follow the Animal Resource Centre at McGill University procedures (protocols: 2011-5864, 2014-7514, and 2001-4830). These experiments comply with the guidelines set by the Canadian Council of Animal Care.

Note that full information on the approval of the study protocol must also be provided in the manuscript.

Human research participants

Policy information about studies involving human research participants

Population characteristics	The patient derived xenografts used in this study were previously developed and reported:<br>Patients with breast cancers (GCRC (DOI: 10.1038/s42003-020-1042-x) and CRC (DOI: 10.1038/s41467-019-10138-8) (Figure 8e-h, S9a-c) and Patients with lung cancer brain metastases (Fig S7) (DOI: 10.1093/neuonc/noab002).<br>GCRC2080: Established from a HER2+ primary breast cancer, IDC (NOS) histology, grade 3. ER neg, PR neg.<br>CRC-132: Established from a primary HER2+ breast cancer, the patient was pre-treated with systemic chemotherapy and resisted to Trastuzumab.<br>GCRC1735: Established from primary Basal breast cancer, IDC (NOS) histology, grade 3, ER neg, PR neg, HER2 borderline, received systemic chemo.<br>GCRC1915: Established from primary Basal breast cancer, IDC (NOS) histology, grade 3, ER neg, PR neg, HER2 neg, rececived systemic chemo.<br>GCRC1963: Established from primary Basal breast cancer, IDC (NOS) histology, grade 3, ER neg, PR neg, HER2 neg, no prior chemo.<br>GCRC1986: Established from a liver recurrence of a Basal breast cancer, IDC (NOS) histology, ER 0.8, PR neg, HER2 neg, systemic chemo received.
Recruitment	Recruitment was performed at both the McGill University Health Center and Jewish General Hospitals, following the protocols established with the ethics below.

Ethics oversight	Patient-derived xenografts (PDX) were developed from tumor material graciously donated by patients who provided informed consent. The procedures were in accordance with the McGill University Health Center research (SUR-99-780); Jewish General Hospital ethics boards for 1) JGH breast biobank (protocol # 05-006) and 2) the Generation of patient-derived material protocol (14-168); McGill University and the Montreal Neurological Institute-Hospital ethics boards (MNIH) (IRB # 2018-4150); and the McGill University Animal Care Committee (2014-7514, and 2001-4830) protocols.

Note that full information on the approval of the study protocol must also be provided in the manuscript.

Flow Cytometry

Plots

Confirm that:

- [x] The axis labels state the marker and fluorochrome used (e.g. CD4-FITC).
- [x] The axis scales are clearly visible. Include numbers along axes only for bottom left plot of group (a 'group' is an analysis of identical markers).
- [x] All plots are contour plots with outliers or pseudocolor plots.
- [x] A numerical value for number of cells or percentage (with statistics) is provided.

Methodology

Sample preparation	Cell lines used for flow cytometry : MT4788 (derived from MMTV-Middle T -transgenic mouse), MDA-MB-231 and BT474 human cell lines. Cell lines were treated with indicated drug concentrations and time described in figure legends, washed, trypsinized and then stained. Detailed staining and sample preparation is outlined in methods section under appropriate subheadings.
Instrument	BD LSRFortessa Analyzer
Software	Collection was done with the BD FACSDiva TM v8.0.1 Software. Analysis was done using FlowJo 10 Software by Tree Star.
Cell population abundance	We did not perform flow-assisted cell sorting, therefore cell population abundance is not relevant to our study.
Gating strategy	For MitoSOX experiments gating was performed: 1) to avoid debris FSC-A vs SSC-A plot 2) Single cells (FSC-A vs FSC-H plot) 3) Live cells (DAPI negative) 4) MitoSox vs FSC-H [gates based on unstained and MitoSox single stained controls]. For Annexin V and PI experiments: gating was as follows: 1) to avoid debris on FSC-A vs SSC-A plot 2) Gated Annexin V-AF647 vs. PI, the quadrants were based on single stained controls of both. For BrdU incorporation assay gating was performed: 1) to avoid debris FSC-A vs SSC-A plot 2) Single cells (FSC-A vs FSC-H plot) 3) BrdU-AF647 vs FSC-A, [BrdU positive gate was set based on unstained controls]. For DCFDA experiments gating was : 1) to avoid debris FSC-A vs SSC-A plot 2) Single cells (FSC-A vs FSC-H plot) 3) Live cells (PI negative) 4. DCFDA vs FSC-H [gates based on unstained and DCFDA single stained controls].

- [x] Tick this box to confirm that a figure exemplifying the gating strategy is provided in the Supplementary Information.
